# Findings of PTSD-specific deficits in default mode network strength following a mild experimental stressor

**DOI:** 10.1038/s44277-024-00011-y

**Published:** 2024-06-17

**Authors:** Christopher L. Averill, Lynnette A. Averill, Teddy J. Akiki, Samar Fouda, John H. Krystal, Chadi G. Abdallah

**Affiliations:** 1 Menninger Department of Psychiatry and Behavioral Sciences, Baylor College of Medicine, Houston, TX USA; 2 Michael E. DeBakey VA Medical Center, Houston, TX USA; 3 National Center for PTSD – Clinical Neurosciences Division, US Department of Veterans Affairs, West Haven, CT USA; 4 Department of Psychiatry, Yale University School of Medicine, New Haven, CT USA; 5 Core for Advanced Magnetic Resonance Imaging (CAMRI), Baylor College of Medicine, Houston, TX USA; 6 Department of Psychiatry, Stanford University, Stanford, CA USA; 7 Department of Psychiatry, Duke University School of Medicine, Durham, NC USA

**Keywords:** Predictive markers, Post-traumatic stress disorder

## Abstract

Reductions in default mode (DMN) connectivity strength have been reported in posttraumatic stress disorder (PTSD). However, the specificity of DMN connectivity deficits in PTSD compared to major depressive disorder (MDD), and the sensitivity of these alterations to acute stressors are not yet known. 52 participants with a primary diagnosis of PTSD (*n* = 28) or MDD (*n* = 24) completed resting-state functional magnetic resonance imaging immediately before and after a mild affective stressor. A 2 × 2 design was conducted to determine the effects of group, stress, and group*stress on DMN connectivity strength. Exploratory analyses were completed to identify the brain region(s) underlying the DMN alterations. There was significant group*stress interaction (*p* = 0.03), reflecting stress-induced reduction in DMN strength in PTSD (*p* = 0.02), but not MDD (*p* = 0.50). Nodal exploration of connectivity strength in the DMN identified regions of the ventromedial prefrontal cortex and the precuneus potentially contributing to DMN connectivity deficits. The findings indicate the possibility of distinct, disease-specific, patterns of connectivity strength reduction in the DMN in PTSD, especially following an experimental stressor. The identified dynamic shift in functional connectivity, which was perhaps induced by the stressor task, underscores the potential utility of the DMN connectivity and raises the question whether these disruptions may be inversely affected by antidepressants known to treat both MDD and PTSD psychopathology.

## Lay Summary

To study any differences between PTSD and depression in the way the brain talks with itself in its default mode when not doing any particular thing, we did MRI brain scans with 52 people with Depression, but only some had PTSD. We found that mild emotional stress may briefly reduce default mode strength in PTSD, but not in depression. This might help researchers better understand the impact of stress and trauma on the brain.

## Introduction

Substantial evidence suggests that major depressive disorder (MDD) and posttraumatic stress disorder (PTSD) are both associated with neural atrophy and synaptic alterations within cortical and limbic circuits implicated in the regulation of mood, cognition, and behavior [1–4]. However, the field has yet to unravel the neurobiological mechanisms underlying these disorders in a fine-grained manner. This has made it difficult to discover potential biomarkers for this constellation of symptoms that could be leveraged for novel diagnostic or treatment approaches and the practice of precision medicine. Advances in neuroimaging methods and analyses over the past two decades have led to a paradigm shift in clinical neuroscience research, with early neuroimaging studies in depression and PTSD identifying a number of abnormal regions of interest (ROI), which were then integrated into seed-based, and more recently, network-based approaches, which generally suggested an association between stress-related psychopathology and alterations in functionally distinct brain networks with high intrinsic connectivity—dubbed *intrinsic connectivity networks* (ICNs; [5, 6]). ICNs are communities of nodes within the whole-brain functional connectome that can be reliably identified across many brain states (including rest) [7], and that share a distinct functional and spatial consistency with the rest of their particular subsystem [8].

The default mode network (DMN) was the first [9] and is one of the most robustly identified ICNs [6]. This network is most active when the mind is at rest and not focused on specific tasks or engaged in any particular processes [6]. The DMN is thought to be implicated in self-referential thought and introspection, and may be particularly vulnerable to stress, compared to other networks [5, 10–12]. Studies of DMN alterations in MDD have resulted in mixed findings and often have focused on the ruminative symptoms associated with this pathology [13]. The first study focusing on the DMN in MDD reported increased connectivity [14], although similar studies found both increases and decreases [15, 16]. Meta-analyses have reported increased DMN connectivity in MDD, though based only on a few studies [13, 17]. A recent paper summarizing 38 studies of DMN alterations in MDD noted 18 studies reported increases, 8 decreases, 7 both increases and decreases, and 5 no significant changes [18]. Given the significant overlap in symptoms and the high rates of comorbidity between MDD and PTSD, it is both interesting and important to consider what similarities and/or differences may exist in the DMN relative to these diagnoses and associated symptoms.

Growing evidence suggests that alterations within or between constituent DMN structures may relate to avoidant, dissociative, and intrusive symptoms of PTSD [5]. A recent functional connectivity study demonstrated a reduction in DMN network-restricted strength (NRS), a validated measure of overall connectivity for all nodes within a network [19], in PTSD compared to trauma-exposed controls [12]. Other work investigating brain regions within the DMN has found evidence of reduced functional connectivity [20–25], disrupted equilibrium *between* the DMN and other ICNs [22] and differentially disrupted equilibrium *within* subsystems of the DMN itself [23], and localized gray matter abnormalities [26–28]. These findings may prove to be clinically informative, as some studies have already shown that either pharmacotherapy or psychotherapy treatment response may influence these findings [18] and/or help to normalize or overcome these alterations [29–32].

While reproducible evidence implicates the DMN in MDD and PTSD, it is not yet clear whether these alterations are disorder-specific or are shared amongst stress-related disorders. Here we aim to address this question by directly investigating DMN connectivity in PTSD compared to MDD. An additional gap in the literature is whether these disruptions of DMN connectivity are trait- or state-dependent, i.e., stress-induced network shifts. To examine this, we investigated whether DMN distinct connectivity patterns would emerge between PTSD and MDD after exposure to an in-scanner mild affective stressor. We hypothesized that individuals with PTSD will show a distinct pattern of connectivity in the DMN following a visual stressor with negative and arousing valence.

## Patients and Methods

### Participants

Participants data were extracted from a data repository at the Clinical Neuroscience Division, National Center for PTSD. The repository collects standardized behavioral and neuroimaging data from participants in ongoing clinical trials, which often target a Veterans population with chronic and treatment-resistant disorders. Baseline pretreatment data were utilized in this report. All participants provided written informed consent. The study procedures were approved by the Institutional Review Board in the Human Research Protection Program at Yale University and all procedures adhered to institutional guidelines and the Declaration of Helsinki. Participants were included in the current study if they were between the age of 21 and 68 years and met primary diagnostic criteria of posttraumatic disorder (PTSD; *n* = 28) or major depressive disorder (MDD; *n* = 24). To account for the high depression comorbidity in our PTSD population, only PTSD participants with secondary depression diagnosis were included in our PTSD group. The MDD group does not meet criteria for PTSD. The Structured Clinical Interview for DSM IV (SCID-IV; [33]; MDD *n* = 10, PTSD n = 14) and MINI International Neuropsychiatric Interview for DSM-5 (MINI-7; [34]; MDD *n* = 14, PTSD *n* = 14) were used to assess for depression and psychiatric comorbidities, and the DSM-IV [35] (n = 15) and DSM-5 [36] (*n* = 13) versions of the Clinician Administered PTSD Scale (CAPS) were used for PTSD diagnosis. The Quick Inventory of Depressive Symptomatology, 16-Item, Self-Report version (QIDS; [37]) was used for depressive symptoms. The Posttraumatic Stress Disorder Checklist versions 4 (*n* = 15) and 5 (*n* = 13) were given to PTSD patients in the original dataset, but an empirical method to crosswalk PCL-4 scores to PCL-5 equivalent scores [38] was used to allow comparison of symptom severity across all patients in the PTSD group using a single PTSD severity value.

Patients were allowed to be on antidepressant treatments, but they were excluded from analysis if the urine toxicology test on the day of their scan had been positive for benzodiazepines, cannabis, cocaine, opioids, or amphetamines. To enhance the generalizability of the findings, psychiatric comorbidities were permitted and are reported in the results section. All participants had completed a magnetic resonance imaging (MRI) scanning session and were carefully screened to confirm they did not have MRI contraindications. Participants were excluded if they had moderate-to-severe traumatic brain injury, epilepsy, brain tumors, or other gross neurological disorders.

### Neuroimaging

**Fig. 1:**
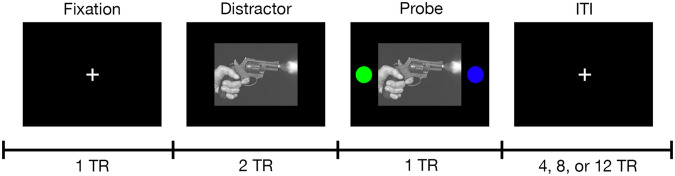
Overview of Negative/Neutral (“Stressor”) Task fMRI Paradigm. During each trial, participants were shown a fixation cross, a negatively or neutrally valanced distractor image from the International Affective Picture System (IAPS), or a distractor image with isoluminant colored circles. While the circles were displayed, the participant used one of two buttons in an MR safe optical response system to indicate the side of the screen on which they saw the blue circle. Each trial was separated by a jittered intertrial interval that was randomized during development but not from scan to scan. Onset times are specified in TR in this figure, where TR = 700 ms. Because IAPS images are not in the public domain, an example image has been used with permission under the Unsplash.com license. Gun photo credit: Tsvetoslav Hristov (@tsvetoslav) on Unsplash.

Structural MRI and multiband functional MRI (fMRI) were acquired in agreement with the Human Connectome Project (HCP) recommendations [39], on a 3 Tesla magnet. Following a 3-plane localization, T1-weighted (MPRAGE; TR = 2400 ms; TE = 2.01 ms; TI = 1000 ms; Flip = 8°; FOV = 256 mm; Voxel = 0.8 × 0.8 × 0.8 mm) and T2-weighted (SPACE; TR = 2400 ms; TE = 565 ms; FOV = 256 mm; Voxel = 0.8 × 0.8 × 0.8 mm) images were acquired to aid in brain extraction, tissue segmentation, and coregistration. Two resting state fMRI scans were acquired immediately before and after a previously validated cognitively-engaging affective task paradigm in which the participant is presented with aversive and neutral images selected from the International Affective Picture System [40]; see Fig. 1). The fMRI sequence of each run was as follow: TR = 700 ms; TE = 31.0 ms; Flip = 55°; Multiband = 6; PE = AP; FOV = 210 mm; Voxel = 2.5 × 2.5 × 2.5 mm; 650 frames. One single-band reference image was collected at the beginning of each fMRI sequence using the same parameters. Immediately preceding the fMRI acquisition block, two single-band spin echo field maps were acquired in opposing phase encoding directions for use in preprocessing of the fMRI (TR = 7220 ms; TE = 73.0 ms; Flip = 90°; Refocus = 180°; FOV = 210 mm; Voxel = 2.5 × 2.5 × 2.5 mm).

In the scanner, immediately between two resting-state fMRI scans, patients completed both a brief task training followed by the task fMRI paradigm. Figure 1 provides a brief overview of the task paradigm. Table S1 provides the list of images used in the task. The task consists of 48 trials (24 each of negative and neutral images); many of the aversive images were extremely graphic. The order of negative and neutral trials was pseudorandomized at design but not per patient [40].

To exert mild experimentally arousing negative stressors, we utilized an affective task that was previously shown to induce increased emotionally aversive and arousing affects following negative pictures compared to neutral images in both healthy and mental illness patients [40]. The International Affective Picture System (IAPS) [41] images were adjusted by the original task authors to be consistent in luminance, contrast, figure-ground relationships, spatial frequency, and color to reduce the likelihood of differential activations based on factors other than image content (i.e., aversive vs neutral). The IAPS creators used a 9-point affective rating process to create a norm-reference value for the dimensions of valence (e.g., happy to sad; higher values indicate more positive content), arousal (e.g., excited to sleepy; higher values indicate stronger intensity of evoked emotion), and dominance (e.g., in control to dominated; higher values indicate feeling more in control). These reference values along with content descriptions and visual review allow investigators to select images from the IAPS database most relevant to the experiment they are designing. In this paradigm, the 24 negative images have mean IAPS ratings of valence = 2.298 (SD = 1.498), arousal = 6.253 (SD = 2.305), and dominance=3.404 (SD = 2.177). These images evoke negative, intense, dominating feelings. The 24 neutral images have mean IAPS ratings of valence=4.903 (SD = 1.1734), arousal = 2.909 (SD = 1.878), and dominance = 5.870 (SD = 1.980). These images evoke neutral, boring, in-control feelings. The aversive images were considerably more negative in nature and more arousing in the IAPS norm reference sample, adding additional evidence that a level of affective stress is elicited by the task.

We used the HCP’s minimal preprocessing pipeline (github.com/Washington-University/Pipelines) to preprocess our scans as described in previous studies [42–45]. Briefly, this included FreeSurfer automatic segmentation and parcellation of T1- and T2-weighted scans, and for the fMRI, slice-time, and motion correction, removal of five initial volumes, intensity normalization, brain masking, and registration to T1w (subject space) and MNI152 template (standard space). The cortical ribbon was projected into a standard 2 mm grayordinate (CIFTI) space. We regressed out motion artifacts (6 degrees of motion, first derivatives, and their squares) and then completed ICA-FIX as implemented by the HCP [46, 47] followed by mean gray ordinate time series regression (MGTR). Image processing and quality evaluation were completed while blinded to demographics and clinical details.

### Default mode network strength

Using the HCP multimodal parcellation (MMP), we divided the cortex into 360 ROIs [48]. A previously validated network-restricted topology approach was used to determine the total DMN connectivity strength [12]. Briefly, we extracted and averaged time series from all grayordinates within each ROI, and calculated the pairwise Pearson correlation coefficients, followed by a Fisher z-transformation to stabilize the variance. The network affiliation of each node was based on a consensus ICN definition specifically designed for the MMP and obtained from a large number of individuals [8]. The hierarchical atlas identifies the following ICNs: the default mode (DMN), central executive (CE), dorsal salience (DS), ventral salience (VS), somatomotor (SM), and visual (VI) networks. The DMN was used in our analyses, and the nodes belonging to the DMN are reported in Table S2. All six networks are depicted for reference in Figure S1. DMN strength is calculated as the mean connectivity between all nodes within the DMN [12, 49].

### Statistical analysis

We used the Statistical Package for the Social Sciences (SPSS, version 28) for conducting the statistical analyses. Probability plots and test statistics were used to examine the distribution of outcome measures. Transformations and non-parametric tests were used as necessary. Estimates of variation are provided as the standard error of the mean (SEM). Significance was set at *p* ≤ 0.05, with 2-tailed tests. Independent *t*-test and Chi-squares were used to compare demographics and clinical data across groups. Mann-Whitney test was used to compare motion during scans across groups. Repeated-measure general linear models (GLM) were constructed to determine the group effect (PTSD vs. MDD), stress effect (baseline vs. post-affective task), and group*stress interaction. To examine potential confounds, we repeated the GLM with various covariates such as head motion, age, sex, depression medication status, comorbid diagnoses. To contextualize our results with our ability to detect certain effect sizes, a power analysis was conducted.

To test the possible effects of scan duration, we first split the pre-stress fMRI in half and calculated the DMN connectivity strength for each half. We then repeated the methods of the primary GLM but looking for effects related to within-session time (first and second half DMN strength) rather than to stress (pre/post task). We did the same for the post-stress fMRI.

## Results

The two groups were well matched for age, sex, handedness, antidepressant medication status, and head motion (Table 1). Current psychiatric comorbidities, enumerated in Table 1, included: generalized anxiety, substance use, alcohol use, panic, obsessive-compulsive, and social anxiety disorders. None of these psychiatric comorbidities significantly differed between the PTSD and MDD groups. Regarding our ability to detect changes in DMN connectivity following a mild stressor, power analysis (see Table 2) suggests we can detect a small effect of stress, a medium effect of group, and a large effect of the interaction of group*stress.

**Table 1:** Demographic and Clinical Characteristics.

	**Full Group**		**PTSD**		**MDD**		**Δ**
**Measure**	***N***	**Mean (SEM)**	***n***	**Mean (SEM)**	***n***	**Mean (SEM)**	***p***
Age	52	41.38 (1.90)	28	42.47 (2.49)	24	40.00 (2.93)	0.50
Head Motion (Pre-Stress)*	52	0.10 (0.01)	28	0.10 (0.01)	24	0.11 (0.01)	0.52
Head Motion (Post-Stress)*	52	0.09 (0.01)	28	0.09 (0.01)	24	0.09 (0.01)	0.60
QIDS-SR16 Total Score	52	12.85 (0.69)	28	13.43 (0.86)	30	12.17 (1.10)	0.36
	***N***	**%**	***n***	**%**	***n***	**%**	***p***
Sex (Male)	35	67	19	68	16	67	0.93
Current Antidepressant	24	46	13	46	11	46	0.97
Handedness (Right)	45	87	23	82	22	92	0.14
Current Comorbid Disorders							
- Substance Use	5	10	3	11	2	8	0.77
- Alcohol Use	7	13	5	18	2	8	0.32
- Generalized Anxiety	15	29	8	29	7	29	0.96
- Panic Disorder	11	21	8	29	3	13	0.16
- OCD	2	4	2	7	0	0	0.18
- Social Anxiety	14	27	8	29	6	25	0.77

*QIDS-SR16* Quick Inventory of Depression Symptomatology-Self Report (16 Item) [59], *Δ* between-group difference, *OCD* obsessive-compulsive disorder. * Relative head motion.

**Table 2:** Power Analysis for the GLM (Cohen’s d for Stress, Group, and Interaction Effects).

Power	Stress Effect	Group Effect	Interaction Effect
80%	0.40	0.56	0.79
85%	0.42	0.60	0.85
90%	0.46	0.64	0.92

### PTSD-specific alterations in default mode network strength

**Fig. 2:**
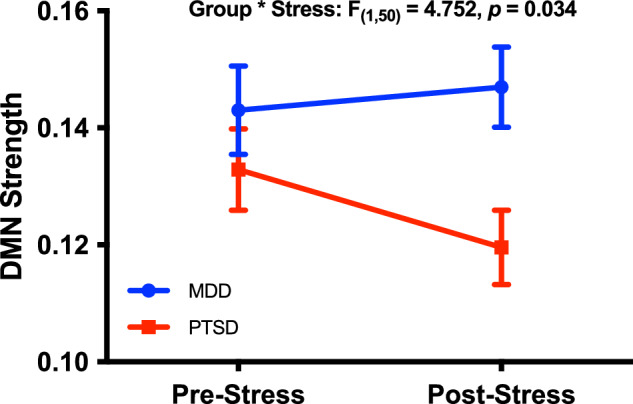
PTSD-Specific stress-induced connectivity decrease within the default mode network. Default mode network (DMN) strength was significantly reduced following the affective stressor in the PTSD group but not MDD. DMN strength rather than z-scores were used in this study to aid in comparison to previous research of DMN connectivity in PTSD.

The primary analysis constructed a full-factorial GLM with DMN strength as the within subject outcome variable. The GLM primarily showed a significant group*stress interaction (F_(1,50)_ = 4.75, *p* = 0.03; Fig. 2), revealing a stress-induced significant 10% reduction in DMN strength in the PTSD group (mean difference = 0.013, SEM = 0.005, *p* = 0.02), with numerical 3% increase in DMN strength in the MDD (mean difference = 0.004, SEM = 0.006, *p* = 0.50) groups. The GLM also demonstrated a significant main effect of group (F_(1,50)_ = 4.35, *p* = 0.04), showing a 13% reduction in DMN strength in the PTSD group compared to the MDD group (mean difference = 0.019, SEM = 0.009). There was no main effect of stress across groups (F_(1,50)_ = 1.392, *p* = 0.244).

Notably, the DMN strength group difference between PTSD and MDD was most robustly evident (18%) following experimental stress (mean difference = 0.027, SEM = 0.009, *p* = 0.005), with only 7% at baseline before the experimental stress (mean difference = 0.010 SEM = 0.010, *p* = 0.33).

### Potential drivers and confounds of the DMN deficits in PTSD post-stress

**Fig. 3:**
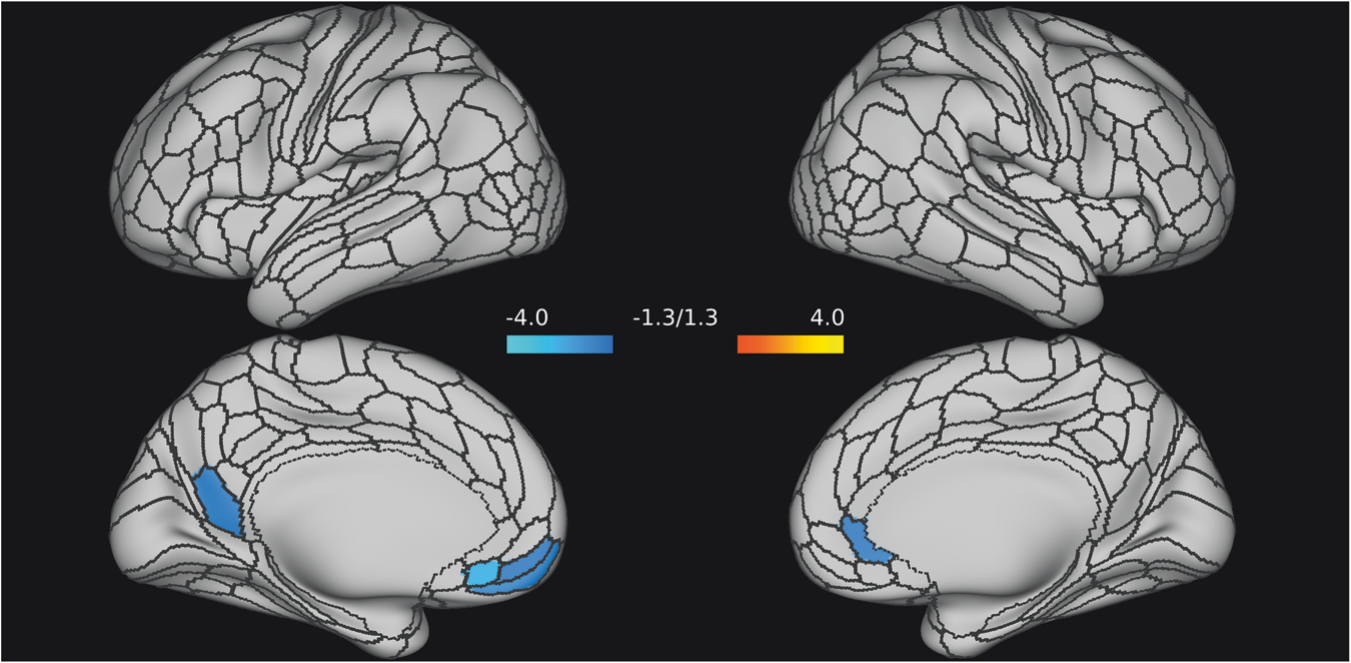
Localization of PTSD-specific stress-induced connectivity changes. Alterations of the Default Mode Network (DMN) connectivity strength are primarily localized in nodes of ventromedial prefrontal cortex (specifically, nodes of the left medial orbitofrontal, and of the right rostral anterior cingulate) and in the left precuneus.

The main analysis showed a significant interaction effect (post-stress difference between MDD and PTSD groups). However, that analysis does not indicate which nodes of the DMN may be implicated. To help contextualize the main results, we explored which cortical regions of the DMN may be driving the difference in DMN connectivity strength post-stress. This nodal analysis of DMN strength between MDD and PTSD groups following experimental stress provided a preliminary indication that the reduction of DMN strength may be driven by connectivity alterations in the midline structure, in particular nodes in the ventromedial prefrontal cortex and the precuneus (see Fig. 3). However, given the large number of nodes within the DMN, these exploratory results do not survive correction for multiple comparisons.

The design of this study could possibly confound stress and time, and the resting state functional connectome may be sensitive to the scan duration [50]. It is reasonable to expect that any effect of scan duration confounding this design would become evident within a given fMRI run. To explore whether the main findings might reflect differential susceptibility to scan duration, we split each of the two fMRI (pre- and post-stress) in half to compare the DMN connectivity strength within each run to check if scan duration effects are observed, and if they are like the observed group*stress interaction of the main analysis.

For the pre-stress fMRI: follow-up GLM showed a significant group*time interaction (F_(1,50)_ = 6.55, *p* = 0.01; Supplementary Fig. S2A), revealing a duration-induced significant 10% increase in DMN strength in the PTSD group (mean difference = 0.014, SEM = 0.006, *p* = 0.01), with numerical 5% decrease in DMN strength in the MDD group (mean difference = 0.007, SEM = 0.006, *p* = 0.27). In the first half of the first rfMRI there was a significant main effect of group (F_(1,50)_ = 5.44, *p* = 0.02), showing a 17% reduction of DMN strength in the PTSD group compared to the MDD group (mean difference = 0.025, SEM = 0.011). There was no main effect of within-session time (F_(1,50)_ = 0.833, *p* = 0.366).

For the post-stress fMRI: follow-up GLM did not find a significant group*time interaction (F_(1,49)_ = 0.149, *p* = 0.70; Supplementary Fig. S2B).

Finally, to evaluate the effect of potential confounds, we ran another follow-up analysis of DMN connectivity group*stress effect with covariates including head motion, age, sex, depression medication status, and other clinical diagnoses like substance and alcohol use disorders, generalized anxiety disorder, panic disorder, OCD, and social anxiety disorder. These did not alter the primary finding of a significant group*stress interaction showing a PTSD-specific reduction of DMN connectivity post-stress (F_(1,39)_ = 7.22, *p* = 0.011).

### The DMN strength alterations are independent of disorder severity

Considering that depression was previously associated with increased connectivity within the DMN [14–16], we examined the possibility that depression symptom severity may have diminished the effects of experimental stress. That is whether higher depression positively correlates with changes in DMN strength following experimental stress. First, consistent the fact that our patient population is often treatment resistance with chronic illness and that all PTSD participants have secondary depression, we found no significant difference in QIDS severity between the PTSD (13.43 ± 4.6) and MDD groups (12.17 ± 5.4). Also, no significant correlation was found between the delta DMN strength (post-stress minus pre-stress) with QIDS severity in either the MDD (*r* = 0.14, *n* = 24, *p* = 0.51) or PTSD group (*r* = 0.03, *n* = 28, *p* = 0.86).

Next, we examined the relationship between changes in DMN strength and PTSD symptom severity in the PTSD group. There was no significant correlation between delta DMN strength and PTSD total severity (*r* = 0.25, *n* = 28, *p* = 0.20). When evaluating the subscale scores, avoidance was found to be positively correlated with delta DMN NRS (*r* = 0.376, *p* = 0.048). However, this finding does not survive correction for multiple comparisons. Reexperiencing (*r* = 0.15, *p* = 0.45), negative alterations of cognition or mood (*r* = 0.34, *p* = 0.08), and arousal (*r* = 0.17, *p* = 0.39) were not correlated with delta DMN strength.

## Discussion

Consistent with the study hypothesis, the results appear to demonstrate a PTSD-specific DMN alteration, which may be a state-dependent abnormality that is exacerbated following exposure to mild stress. These results may suggest that although both MDD and PTSD are stress-related disorders, and despite the high rates of comorbidity and symptom overlap, PTSD may be associated with a unique connectomic signature that is distinct from that of MDD. The seeming PTSD-specific dynamic network shift that we observed in the DMN may aid in our understanding of both normal brain function and the pathways through which stress and trauma can differentially augment patterns of connectivity. Findings such as these are important as any distinct biomarkers that differentiate diagnoses or constellation of symptoms from one another, can be useful in informing novel drug development and precision medicine efforts. Critically, the findings also suggest that the mixed results in the literature may be explained, at least in part, by state-differences at the time of imaging acquisition. PTSD is a highly heterogeneous disorder [51], and, while there are likely trait effects of stress-related psychopathology on the brain, these can be masked by opposing state effects [44, 45, 52].

In the present findings, the main effect of group appears to be driven by the interaction with stress. Post-stress MDD patients with PTSD appeared to exhibit reduced DMN connectivity strengths compared to post-stress patients with MDD only. That is to say, prior to stress, the pathology underlying these comorbid disorders did not differ significantly (trait-like presentation), but when acutely stressed (state-like presentation), the PTSD group appeared to exhibit an exaggerated, state-induced reduction of within-DMN connectivity. While mounting evidence suggests alterations within or between constituent DMN structures may relate to avoidant, dissociative, and intrusive symptoms of PTSD [5], the results beg the question whether hyperarousal and avoidance inherent and unique to PTSD relative to MDD, may relate to heightened state arousal in PTSD overcoming the trait-like features shared with MDD. Current symptom severity seems not to be related to the stress-induced reduction of DMN NRS, indicating it may be a trait rather than a state feature. However, given the slight correlation with avoidance subscale, future studies should further investigate the question of state vs trait dependence to better understand any exacerbating effect that symptom profile (especially avoidance) may have upon this trait-dependent alteration. It is also possible that patients with MDD undergo similar network shifts *during* acute stress, but return to baseline rapidly, whereas the hyperarousal, exaggerated startle, and anxiety symptoms of PTSD may significantly extend the state-induced shift, slowing return to baseline connectivity.

Though the affective stressor task is not intended to be traumatic, the negative images (which include many images of dead or mutilated bodies) could cause some level of distress. This might be particularly likely for individuals who have witnessed comparable graphic events in a combat zone or during other traumatic experiences, or for whom the images evoke similar emotional or cognitive responses to those they experienced at the time of an original trauma. PTSD is characterized by reexperiencing symptoms that include intrusive thoughts and memories related to one’s trauma(s), negative cognitions and mood (e.g., guilt, shame, blame), and avoidance of thoughts and feelings as well as avoidance of people, places, and activities that may serve as a reminder of past or ongoing trauma(s) [53]. It is plausible that the exposure to the aversive images during the task was triggering for patients with PTSD, either initiating or exacerbating these symptoms while in the scanner. Although negative cognitions toward oneself, others, and the world are interoceptive in nature, intrusive recollections and ruminations are highly emotional and cognitively engaged experiences. If the stressor is triggering distressing thoughts and memories, it may lead to a shift away from the more mundane “resting” type thoughts, accompanied by the dynamic network shift we observed in the DMN in the present sample of patients with PTSD.

Another finding in this work provides further support to the growing evidence of the complexity and multidimensionality of the DMN. Though this analysis did not survive correction for multiple comparisons and requires replication, our findings warrant further investigation as they suggest a possible distinct pattern of alterations across the DMN in separate constituent regions, namely nodes of the left medial orbitofrontal, right rostral anterior cingulate, and of the left precuneus. The DMN has been described as a heterogeneous network system comprised of at least two distinct subsystems that interact through a core set of ‘hubs’ or cardinal brain regions [11, 23]. One of these two major subsystems, which we will call the anterior DMN subsystem, is comprised primarily of the cingulate cortex and mPFC and is thought to be active during self-referential thought, mentalizing, affective decisions, and socially-based thoughts about the self and others [11, 54, 55]. A second major subsystem, comprised of structures including the precuneus and medial temporal lobe), which we will call the posterior DMN subsystem, is thought to be engaged when decisions are grounded in a mental scene based broadly on learning and memory [11, 23, 54]. The precuneus is also involved in the integration of information and the forming of a gestalt based on perception of the environment, cue reactivity, mental imagery strategies, episodic memory retrieval, and affective responses to pain [56, 57] – which potentially could include traumatic memory elaboration and re-experiencing [57] during exposure to an affective stressor.

The functional connectome is sensitive to brain activity and mental states, providing investigators with clues about cohesive activations and network activity [7]. However, it is also sensitive to various confounding factors including length of scans, heterogeneity in shared and unshared signals, and amplitude variations within and between individuals [50, 58–61]. Further, the MRI environment and scanning itself can be stressful and fatiguing for participants [62], which may differentially alter mental states and network connectivity even within individual fMRI runs. The observation in this study of PTSD-specific DMN connectivity changes within full fMRI runs before and after a stressor, while potentially important for further study, does not address underlying DMN connectivity shifts within each fMRI that may either undermine or explain the observed reduction. We attempted to address this concern by splitting each run in half and comparing the DMN strength of each half within rather than between runs. If significant DMN connectivity shifts within runs were observed to be like the primary finding, confounding of stress (pre-post task) and scan duration effects might reduce confidence in the main result.

We did observe a significant interaction between the group and scan duration in the pre-stress fMRI, manifesting as an *increase* in DMN connectivity in the PTSD group during the run while the MDD group had no significant alteration of DMN connectivity. This PTSD-specific effect of within-session time indicates an increasing drift away from directed thought to a more introspective or mind-wandering state. Comparatively, the MDD group generally maintained their mental state across the pre-stress run, showing only a minor *decrease* in DMN connectivity later in the run, indicating a mild shift outward toward non-DMN tasks. However, in the post-stress fMRI, the effect of within-run fatigue was lost. Neither MDD nor PTSD groups exhibited a significant DMN connectivity shift within this run.

These effects of scan duration appear to be PTSD-specific but do not confound the primary result of the overall reduction of DMN connectivity in the PTSD group following stress. Rather, they at least partly help explain the observed effects of stress. We speculate that the stressor may have altered the attention or susceptibility to scan duration of the PTSD group but not the MDD group. If DMN connectivity in the PTSD group had remained relatively consistent throughout the pre-stress fMRI (no effect of scan fatigue within run), or if their DMN connectivity was observed to increase within the post-stress fMRI (no effect of stress on scan fatigue within run), then we would not observe the same overall reduction of DMN connectivity strength pre-post stress; in fact, that situation is exactly what we observed in the MDD group (no effect of scan fatigue within runs, no overall effect of stress).

Elsewhere, we have discussed the possibility that the primary finding could be explained by the stressor directly reducing DMN connectivity following stress (slower return to baseline than the MDD group, increased salient or cognitive processes as PTSD symptoms are triggered by the stressor, etc.). The evaluation of scan duration effects within runs offers important nuance, that there may be a PTSD-specific general pattern of increasing DMN activity during fMRI that is thwarted by exposure to an acute affective stressor resulting in reduced overall DMN activity during fMRI after stress. Future studies should design experimental paradigms to clarify the nature of this potential biomarker.

This study is limited by several factors. First, the data comes from a repository where some participants were assessed with different versions of the CAPS over time (i.e., CAPS for DSM IV or DSM 5). The same challenge exists with the variability in assessment for depression (SCID vs MINI). There is a lack of information about the chronicity of trauma exposure/symptomology, early life stress, and duration of illness which may result in compounding neural complications. Another limitation is that we lack longitudinal or premorbid baseline scans, so cannot investigate in this sample whether the observations are likely to be a predisposing or subsequent factor with respect to PTSD [63, 64]. The heterogeneity of nodal connectivity within the DMN may also be concerning, as group means might not reflect the variety of connectivity patterns in the actual data. Reference [60] Finally, some findings did not survive analysis for multiple comparisons so must be interpreted with caution and replicated in larger, more diverse, and well-characterized samples.

In contrast to these limitations, the study also benefits from several strengths, including a broad range of ages so results may be generalizable across the lifespan. Another strength of the study is that the sample was well-matched on various dimensions, potentially yielding a clean and relatively interpretable signal. The design is also a strength. Comparing patients with and without PTSD when all participants meet the criteria for MDD allowed us to provide some of the first potential evidence of disease specificity (while maintaining the generalizability considering the high comorbidity in PTSD) regarding altered network connectivity in PTSD, and the use of resting fMRI immediately before and after an affective stressor allowed us to evaluate the effect of and interaction with stress, not only group effects.

## Conclusion

Accurate identification of DMN abnormalities could inform our understanding of the impact of stress and trauma on the brain and resulting behavior, aiding in precision medicine (i.e., rational treatment development, and target validation) [65]. Future research aiming to replicate and extend these findings would benefit from increased diversity in the sample; categorization of trauma-type and chronicity; perceived current stress load; examination of the likely dynamic relationship between ICNs; and careful evaluation of the role of PTSD symptom clusters to further evaluate their impact. Future works might also consider: collecting psychophysiological measures during the scans to help determine the level of physiological arousal during experimental stressors; investigating differences between similar groups during stressors; probing for issues related to heterogeneity; exploring the effect of the scan itself (e.g., duration, fatigue [50], scanner as a stressor [62], etc.); and evaluating the possible impact on DMN connectivity from trauma-exposure and subthreshold PTSD symptoms in MDD patients.

In this study, the PTSD group demonstrated alterations characterized by decreased strength in the DMN, relative to those with MDD. Taken together, the results of this mechanistic study have allowed us to demonstrate stress-induced dynamic network shifts related to the DMN, which could potentially override network pathology in patients with PTSD.

## Supplementary information

Supplemental Material

## Data Availability

Data available upon reasonable request to the corresponding author, Dr. Abdallah, and following completion of a data use agreement limiting the use of shared data and prohibiting re-identification of subjects.
