# Supplementary material for: Findings of PTSD-specific deficits in default mode network strength following a mild experimental stressor
Source: NPP Digit Psychiatry Neurosci. 2024 Jun 17;2:9. doi: 10.1038/s44277-024-00011-y (PMC11197271; doi:10.1038/s44277-024-00011-y)
Supplement: Supplementary file 1 — Supplemental Material [file 44277_2024_11_MOESM1_ESM.docx]

**Supplementary Materials for:** Averill CL, Averill LA, Akiki TJ, Fouda S, Krystal JH, Abdallah CG. Findings of PTSD Specific Deficits in Default Mode Network Strength Following a Mild Experimental Stressor.

**Figure S1.** **Reference Image for Community Affiliation of the Nodes.** This panel demonstrates the cortical network nodal affiliations based on the Akiki-Abdallah cortical hierarchical atlas (6 modules; AA-6),[1] including the default mode (DM), central executive (CE), dorsal salience (DS), ventral salience (VS), sensorimotor (SM), and visual (VI) networks. Figure adapted with permission by the Emerge Research Program (<http://emerge.care>).


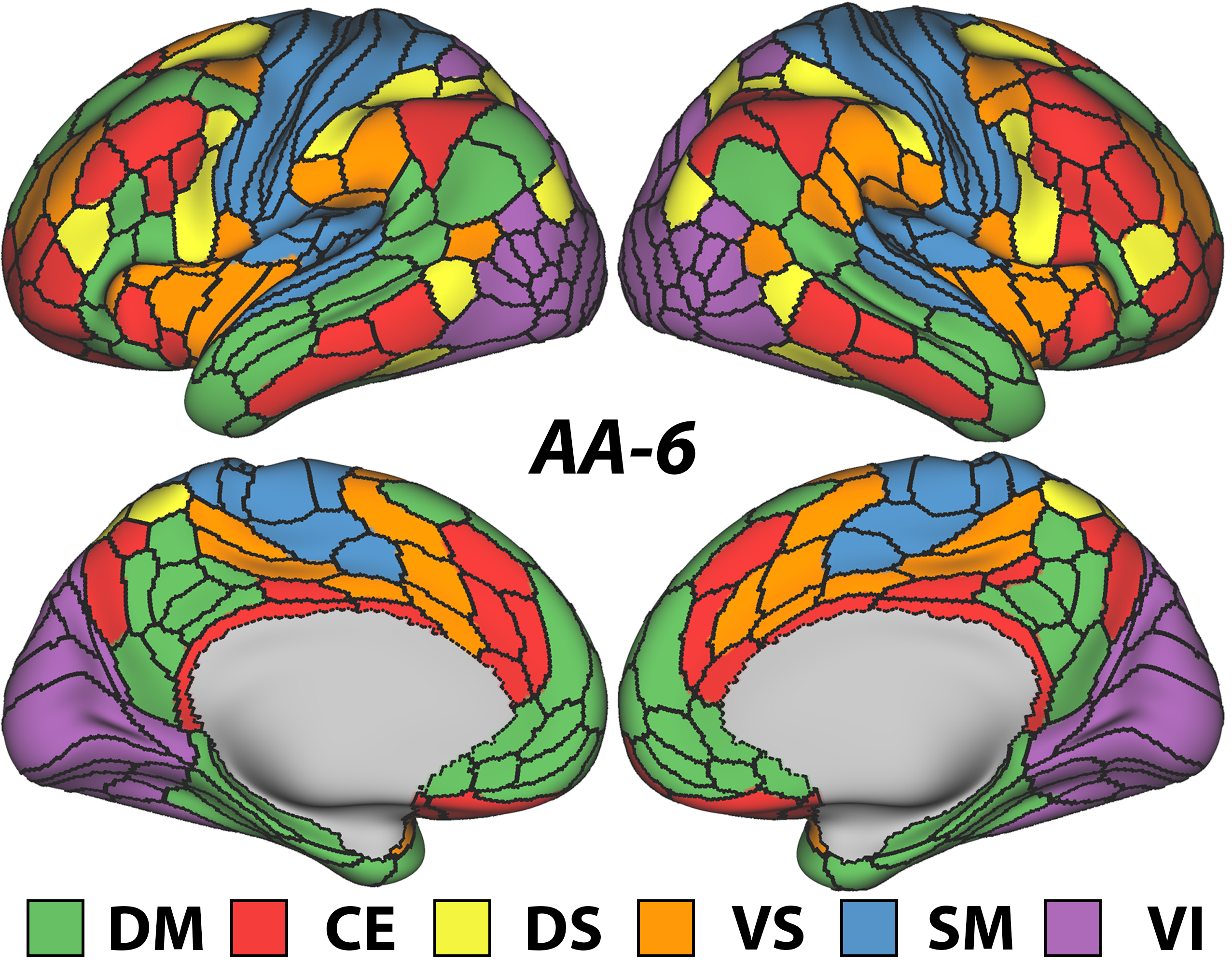


**Figure S2.** **PTSD-Specific Effect of Time on Default Mode Network (DMN) Connectivity Strength Within Individual fMRI Sessions.** This figure details the interaction of within-session time and group on DMN connectivity strength across each of the pre and post stress resting state fMRI sessions (first half vs second half of each scan). **Panel A:** Pre-stress the PTSD group DMN strength increased significantly; the MDD group did not exhibit a significant change of DMN strength. **Panel B:** Post-stress, neither group exhibited a significant change in DMN connectivity.

**
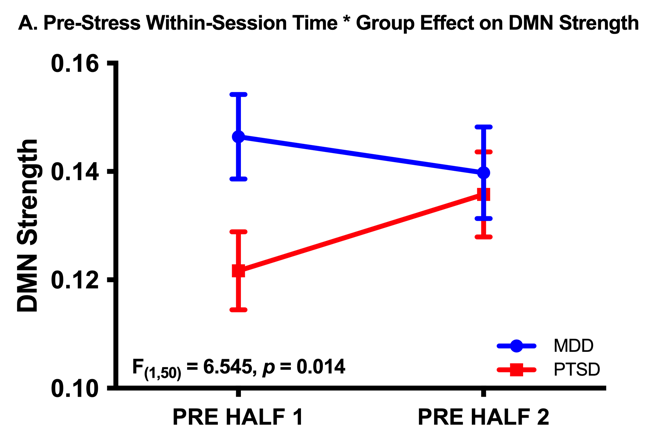

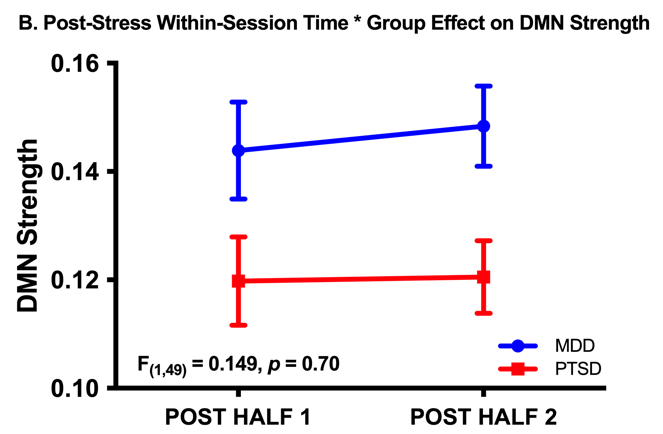
**

**Table S1. Photos used in fMRI task from the International Affective Picture System (IAPS)**

| **Trial** | **Group** | **IAPS ID** | **Description*** | **Trial** | **Group** | **IAPS ID** | **Description*** |
| --- | --- | --- | --- | --- | --- | --- | --- |
| 1 | Neg | 6250 | AimedGun | 25 | Neut | 7190 | Clock |
| 2 | Neut | 7009 | Mug | 26 | Neut | 5390 | Boat |
| 3 | Neg | 6230 | AimedGun | 27 | Neut | 7035 | Mug |
| 4 | Neg | 3100 | BurnVictim | 28 | Neg | 3400 | SeveredHand |
| 5 | Neut | 7060 | TrashCan | 29 | Neut | 7820 | Agate |
| 6 | Neut | 7020 | Fan | 30 | Neg | 1114 | Snake |
| 7 | Neg | 3168 | Mutilation | 31 | Neg | 9410 | Soldier |
| 8 | Neut | 7175 | Lamp | 32 | Neg | 3063 | Mutilation |
| 9 | Neg | 9040 | StarvingChild | 33 | Neut | 7056 | Tool |
| 10 | Neut | 2570 | Man | 34 | Neg | 3010 | Mutilation |
| 11 | Neg | 9594 | Injection | 35 | Neg | 3261 | Tumor |
| 12 | Neg | 3069 | Mutilation | 36 | Neg | 3101 | BurntFace |
| 13 | Neut | 7059 | Keyring | 37 | Neg | 3022 | Scream |
| 14 | Neut | 7110 | Hammer | 38 | Neg | 9571 | Cat |
| 15 | Neg | 3102 | BurnVictim | 39 | Neut | 7050 | HairDryer |
| 16 | Neg | 3068 | Mutilation | 40 | Neg | 2661 | Baby |
| 17 | Neut | 5130 | Rocks | 41 | Neg | 2730 | NativeBoy |
| 18 | Neut | 7002 | Towel | 42 | Neut | 7150 | Umbrella |
| 19 | Neut | 7480 | ElderlyMan | 43 | Neut | 7705 | Cabinet |
| 20 | Neut | 2280 | Boy | 44 | Neg | 3220 | Hospital |
| 21 | Neg | 9253 | Mutilation | 45 | Neg | 1270 | Roach |
| 22 | Neut | 7140 | Bus | 46 | Neut | 7130 | Truck |
| 23 | Neg | 3000 | Mutilation | 47 | Neut | 5534 | Mushroom |
| 24 | Neut | 2440 | NeutGirl | 48 | Neut | 5532 | Mushroom |

*Description provided by IAPS.

**Table S2. Nodes affiliated with the Default Mode Network**

| **Network** | ***N*** | **Node Labels** (See Glasser, Coalson [2]) |
| --- | --- | --- |
| Default Mode (DM) | 96 | R_SFL, R_PCV, R_STV, R_7m, R_POS1, R_v23ab, R_d23ab, R_31pv, R_a24, R_10r, R_47m, R_8Ad, R_9m, R_8BL, R_9p, R_10d, R_45, R_47l, R_9a, R_10v, R_10pp, R_47s, R_EC, R_PreS, R_H, R_PeEc, R_STGa, R_A5, R_PHA1, R_STSda, R_STSdp, R_STSvp, R_TGd, R_TE1a, R_TF, R_TPOJ1, R_PGi, R_PHA2, R_31pd, R_25, R_s32, R_TGv, R_STSva, L_55b, L_PSL, L_SFL, L_PCV, L_STV, L_7m, L_POS1, L_v23ab, L_d23ab, L_31pv, L_a24, L_d32, L_p32, L_10r, L_47m, L_8Av, L_8Ad, L_9m, L_8BL, L_9p, L_10d, L_44, L_45, L_47l, L_IFSp, L_9a, L_10v, L_10pp, L_47s, L_EC, L_PreS, L_H, L_ProS, L_PeEc, L_STGa, L_A5, L_PHA1, L_PHA3, L_STSda, L_STSdp, L_STSvp, L_TGd, L_TE1a, L_TF, L_TPOJ1, L_PGi, L_PGs, L_PHA2, L_31pd, L_25, L_s32, L_TGv, L_STSva |

**References (Supplement):**

1. Akiki, T.J. and C.G. Abdallah, *Determining the Hierarchical Architecture of the Human Brain Using Subject-Level Clustering of Functional Networks.* Sci Rep, 2019. **9**(1): p. 19290.

2. Glasser, M.F., et al., *A multi-modal parcellation of human cerebral cortex.* Nature, 2016. **536**(7615): p. 171-178.
